# No detection of tick-borne encephalitis virus RNA in blood, urine or saliva of hospitalised immunocompetent tick-borne encephalitis patients

**DOI:** 10.1371/journal.pone.0305603

**Published:** 2024-06-24

**Authors:** Hanne Quarsten, Åshild K. Andreassen, Katrine M. Paulsen, Maria J. Diekmann, Randi Eikeland, Rita Helleren, Tomas Bergström, Sølvi Noraas, Åslaug R. Lorentzen

**Affiliations:** 1 Department of Medical Microbiology, Sørlandet Hospital Trust, Kristiansand, Norway; 2 Division for Infection Control and Environmental Health, Department of Virology, Norwegian Institute of Public Health, Oslo, Norway; 3 The Norwegian National Advisory Unit on Tick-borne Diseases, Sørlandet Hospital Trust, Kristiansand, Norway; 4 Faculty of Health and Sport Science, University of Agder, Grimstad, Norway; 5 Department of Clinical Medicine, Sørlandet Hospital Trust, Kristiansand, Norway; 6 Department of Infectious Diseases, Institute of Biomedicine, Sahlgrenska Academy, University of Gothenburg, Gothenburg, Sweden; 7 Department of Neurology, Sørlandet Hospital Trust, Kristiansand, Norway; University of Bologna / Romagna Local Health Authority, ITALY

## Abstract

Tick-borne encephalitis (TBE) is usually diagnosed based on the presence of TBE virus (TBEV)-specific IgM and IgG antibodies in serum. However, antibodies induced by vaccination or cross-reactivity to previous flavivirus infections may result in false positive TBEV serology. Detection of TBEV RNA may be an alternative diagnostic approach to detect viral presence and circumvent the diagnostic difficulties present when using serology. Viral RNA in blood is commonly detectable only in the first viremic phase usually lasting up to two weeks, and not in the second neurologic phase, when the patients contact the health care system and undergo diagnostic work-up. TBEV RNA has previously been detected in urine in a few retrospective TBE cases in the neurologic phase, and furthermore RNA of other flaviviruses has been detected in patient saliva. In this study, blood, saliva and urine were collected from 31 hospitalised immunocompetent patients with pleocytosis and symptoms of aseptic meningitis and/or encephalitis, suspected to have TBE. We wanted to pursue if molecular testing of TBEV RNA in these patient materials may be useful in the diagnostics. Eleven of the 31 study patients were diagnosed with TBE based on ELISA detection of TBEV specific IgG and IgM antibodies. None of the study patients had TBEV RNA detectable in any of the collected patient material.

## Introduction

Tick-borne encephalitis virus (TBEV) is a positive strand RNA virus in the genus flavivirus usually transmitted to humans by *Ixodes ricinus* ticks, or less frequently by consumption of dairy products from infected domestic animals [1].TBEV infection often occurs in a biphasic manner [2]. The first viremic phase is characterised by flu-like symptoms, which after a shorter period with few symptoms, may be followed by a second phase affecting the central nervous system with symptoms of meningitis and/or encephalitis.

The diagnosis of tick-borne encephalitis (TBE) often relies on the presence of TBEV-specific IgM alone or both IgM and IgG antibodies in serum, as the clinical manifestations are not specific for this disease [3, 4]. The serological tests may be precluded by IgM antibodies induced by recent TBE vaccination or long-lasting IgM from previous asymptomatic or mild infection of TBEV. False positive test results may also arise from cross-reactive antibodies remaining from infection by or vaccination against other flaviviruses such as dengue, Zika, yellow fever or Japanese encephalitis viruses [5, 6]. Testing for TBEV neutralising antibodies in serum will verify the presences of TBEV antibodies, yet antibodies induced by TBE vaccination cannot be excluded. Verification of an uncertain serology requires either detection of a four-fold increase in TBEV antibody titre in serum samples taken a least two weeks apart or TBEV antibodies produced in the cerebrospinal fluid (CSF) determined by a positive CSF/serum antibody index [7, 8]. Due to more travelling and vaccination, antibodies cross-reactivity in TBEV serology tests are rising in the population. Thereby, alternative approaches for diagnosing TBE could be beneficial to circumvent the difficulties with antibody testing.

In the first viremic phase of the disease, TBEV viral RNA present in blood may be detected by molecular methods. In the neurologic phase, when most patients seek medical care due to severe symptoms, TBEV RNA is rarely detected neither in blood nor in CSF [9]. A few cases have been reported where TBEV has been detected in urine from patients in the neurologic phase [10–12]. Persistent viremia and urine shedding of TBEV have been demonstrated for more than six weeks in an immunosuppressed patient with persistent infection [11]. Other flaviviruses have also been detected in patient saliva [13].

The aim of this study was to evaluate if detection of TBEV RNA could be useful in diagnosing TBE in the neurologic phase. Samples of blood, urine and saliva from immunocompetent Norwegian patients hospitalised with suspected aseptic meningitis/encephalitis, were collected and tested by PCR.

## Material and methods

### Patients

Patients hospitalised at Sørlandet Hospital were recruited for the study in the period 18th September 2015 to 8th August 2018. Adult patients with pleocytosis (5–200 cells/mm^3^) suspected to have a viral infection of the central nervous system were invited to participate. The patients typically had symptoms of meningitis as headache, stiff neck, fever, reduced general condition and/or symptoms of encephalitis with neurological dysfunction. All patients answered a questionnaire at the time of study inclusion regarding history of recent tick-bites and erythema migrans, time of first symptoms related to the ongoing disease together with information of infection by or vaccination against dengue, yellow fever and TBE viruses.

### Ethical statements

Norwegian Regional Committees for Medical and Health Research Ethics approved the study (2015/938/REK South-East) and all participants gave a written informed consent.

### Samples and handling

Blood (EDTA and serum), saliva (≥0.5 ml) and urine (≥10 ml) samples were collected from all patients. Preparation of nucleic acids and real-time reverse transcription (RT)-PCR analyses of all samples were performed at the Department of Medical Microbiology, Sørlandet Hospital, usually within one day after admission (range 0–3 days). Native patient samples (serum, saliva and urine) from the patients diagnosed with TBE were stored at -70°C before transportation to the Norwegian Institute of Public Health for RNA isolation and additional real-time RT-PCR analyses.

### Serology

All sera were analysed for TBEV-specific IgM and IgG antibodies at Sørlandet Hospital, using the Enzygnost Anti-TBE virus IgG and IgM ELISA kits (Siemens Healthcare Diagnostics Products GmbH, Marburg, Germany) with the manufacturer´s cut-off level and interpretation criteria. For detection of IgM and IgG in serum, the 98% specific diagnostic cut-offs were OD = 0.229 and 0.235, respectively.

### Isolation of RNA

#### RNA isolation at Sørlandet Hospital

Viral RNA was isolated from 140 μl saliva and urine by QIAamp Viral RNA Mini Kit (Qiagen, Hilden, Germany) and total nucleic acids were isolated from 200 μl EDTA blood by QIAamp DNA Mini kit (Qiagen, Hilden, Germany) as described by the manufacturer’s instructions. Extraction volumes were 60 and 100 μl for samples prepared with the RNA and DNA kit, respectively.

#### RNA isolation at Norwegian Institute of Public Health

Viral RNA was isolated from 140 μl saliva, urine and serum by QIAamp Viral RNA Mini Kit as described by the manufacturer’s instructions and extracted in 60 μl elution buffer. Saliva and urine were 2x (140 μl sample on two spin columns) and 4x (140 μl sample on four spin columns) concentrated by extracting nucleic acids from all columns used for each patient material in 60 μl elution buffer in total.

### Reverse transcription and real-time PCR assays

#### PCR analysis at Sørlandet Hospital

RNA (5 μl each) from saliva, urine and EDTA-blood was reverse transcribed to cDNA by using the qScript^TM^ cDNA synthesis kit (Quanta BioScience Incorporated, Gaithersburg, MD, USA). Samples (5 μl cDNA) were tested by two real-time Taqman PCR protocols (designated TBEV1 and TBEV2, Table 1) [14, 15]. The TBEV1 and TBEV2 PCR amplifies a 69 base pair (bp) in the 3’ non-coding region and 54 bp fragment located on the envelope gene of TBEV, respectively. Protocols were performed using 5μl of cDNA in a 15 μl reaction mixture consisting of 5mM MgCl_2,_ 0.5 units Uracil DNA-glycosylase (Eurogentec S.A. Seraing, Belgium), LightCycler FastStart DNA master mix (Roche, Mannheim, Germany) with the primer and probe concentrations given in Table 1 and run on a LightCycler 480 with the following thermocycling parameters; 2 min at 40°C followed by 10 min at 95°C and 45 cycles of 15 s at 94°C, 45 s at 60°C and 30 s at 72°C.

**Table 1:** Characteristics and sequences of TBEV-PCR primers and probes used in in this study.

Assay	Target gene	Oligonucleotide sequence (5’-3’)	Primer/probe concentration (μM)	References
*TBEV1*	non-coding region	*F* TGG GCG GTT CTT GTT CTC C	SS 0.5/0.3	14
		*R* TCA CAC ATC ACC TCC TTG TCA GA		
		*P* CTG AGC CAC CAT CAC CCA GAC ACA G		
*TBEV2*	proteinE	*F* GGG AGC GCA AAA CTG GAA	SS 0.5/0.3 NIPH 0.25/0.3	15
		*R* TGA GGA GCC CCA AAT TCA AC		
		*P* AAC GCA GAA AGA C (MGB)		

Abbreviations: *F*: forward primer; *R*: reverse primer; *P*: probe; MGB: minor groove binder

SS: Sørlandet hospital; NIPH: Norwegian Institute of Public Health

#### PCR analysis at Norwegian Institute of Public Health

RNA (5 μl) were reverse transcribed to cDNA by High High-Capacity cDNA Reverse Transcription Kit (Applied Biosystems, Foster city, CA, USA). Samples (3 μl cDNA) were analysed by TBEV2 PCR (Table 1) performed according to Andreassen et al. [15].

## Results

### Serology

A total of 31 hospitalised patients suspected to have viral infection of the central nervous system were included in the study, and 11 of them were classified as TBE cases primarily based on TBEV antibody testing in serum. All TBE cases had detectable TBEV-specific IgM and IgG antibodies (Table 2). Three patients were tested in consecutive sera, and TBE was confirmed by either increase in both TBEV IgM and IgG or increase in TBEV IgG and decrease in IgM levels. The 20 patients classified as non-TBE, except for two with detectable serum TBEV IgG only, had no detectable TBEV antibodies.

**Table 2:** Presence of serum TBEV IgG/IgM antibodies and characteristics of TBE patients.

Patient no.	TBEV IgG/IgM detection	Self-reported symptoms	Additional symptoms from hospital journal	Hospital stay (days)	Time from first symptoms until samples were drawn (days)	Known tick-bite (<10weeks)	Comments
1	+/+	Fever, myalgia	Headache, nausea, stiff neck, light sensitivity	6	24^a^	Yes	TBEV RNA detected in blood drawn 25 days prior to study inclusion.
2	+/+	Fever, myalgia	Headache, nausea, stiff neck, unsteadiness	3	19	Yes	Increase in IgG in samples drawn 6 days apart
3	+/+	Fever, myalgia, headache	Nausea, stiff neck, light sensitivity	7	22	No	
4	+/+	Fever, myalgia, headache	Nausea, stiff neck, light sensitivity, numb left arm, unsteadiness	2	10	Yes	Increase in IgG and decrease in IgM in samples drawn 23 days apart
5	+/+	Fever, myalgia	Nausea, weak left arm, unsteadiness	2	18		
6	+/+	None	Fever, headache, nausea, light sensitive, dizziness, unsteadiness	9	>19	Yes	TBE vaccinated Increase in IgG and IgM in samples drawn 21 days apart
7	+/+	Fatigue, nausea	Headache, radicular pain, weak legs, unsteadiness	17	>30	Yes	
8	+/+	Fever, myalgia	Headache, nausea, stiff neck, numb fingers, unsteadiness	9	17	Yes	
9	+/+	Fever, myalgia, headache	Nausea, light sensitivity, unsteadiness	6	~ 30	Yes	
10	+/+	Fever, myalgia, headache, nausea	Stiff neck, light sensitivity, double vision, discrete findings of myelitis/radiculitis (MR), unsteadiness	24	27	No	
11	+/+	Fever, myalgia, pain	Feeling sick	8	Not known, >7 days	No	

The male to female ratio was 1.75:1 and the median age 44 (range 30–79) years in the TBE patient group.

^a^Patient self-reported the first day of symptoms 24 days before hospitalisation. Incorrect day of first symptoms was likely to be reported, as the TBEV RNA positive blood sample was drawn at the patient’s general practitioner due to high fever and sore throat the day before.

### Characteristics of TBE patients

The TBE cohort had a male to female ratio of 1.75:1, a median age of 44 (range 30–79) years and all patients were immunocompetent. All 11 TBE patients presented with fever, except for one (Table 2). Further, TBE manifested with a diverse set of symptoms as nausea (91%), myalgia (82%), headache (82%), unsteadiness (73%), stiff neck (55%), light sensitivity (55%), numbness/weakness in extremities (36%) and discrete findings of myelitis/radiculitis (18%). Nine out of 11 TBE patients provided samples for the study 17 days or more after the first sign of illness. One patient provided samples 10 days after the first notified symptoms and for one patient information about disease onset was lacking. All but three TBE patients recalled a recent tick-bite.

### Molecular detection

RNA isolated from whole blood, urine and saliva of all study patients was analysed by the TBEV1 and TBEV2 RT-PCR for detection of TBEV RNA at Sørlandet Hospital. No samples were TBEV RNA positive. RNA isolated from serum, 2x concentrated saliva and 4x concentrated urine from the 11 patients classified as TBE cases were again analysed by the TBEV2 RT-PCR at the Norwegian Institute of Public Health. No samples were TBEV RNA positive.

## Discussion

TBEV RNA was not detected by RT-PCR in neither serum, whole blood, saliva nor urine from any of the 11 immunocompetent TBE patients in the second neurologic phase, hospitalised at Sørlandet Hospital, Norway. Concentrated saliva (2x) and urine (4x) samples were analysed to increase the rate of TBEV RNA detection without any success. All TBE patients had both detectable virus-specific IgM and IgG at the time patient samples were analysed by two different TBEV RT-PCR analyses, indicating that most if not all patients were late in the disease course.

Our findings contrast with what reported previously for four TBE patients in the neurologic phase, where TBEV RNA was detected in urine samples [10–12]. Three of the patients presented in these studies were on immunosuppressive drugs and had severe disease courses. One of them had detectable IgG in serum and TBEV RNA in urine at day 49 after disease onset, another had detectable IgM only and TBEV RNA present in urine from day 7–16 and the third had no detectable TBEV antibodies whereas TBEV RNA were present in both CSF and urine at the day of hospitalisation [10–12]. An immunocompetent patient seroconverted during the hospital stay (day 17) and TBEV RNA was detected in urine at day 19 [10]. No TBEV RNA was detected in this patient four days later. It should though be underlined that detection of TBEV RNA in urine is a sporadic event and in most cases the samples are negative [16].

The TBE patients in our study were immunocompetent and late in their disease course thus they all had detectable virus-specific antibodies. Antibodies are significant for viral clearance, as demonstrated by others TBEV RNA in blood is disappearing when TBEV IgM and IgG antibodies are appearing [17]. Along this line, the presence of TBEV-specific IgM is reported to be associated with a mild disease presentation [18] whereas long lasting low levels of virus-specific IgG are associated with severe and prolonged clinical courses [19]. All but two of the TBE patients in our study had hospital stays for nine days or less, consistent with a mild/moderate illness probably due to a well-established immunity against the infection.

A serum sample from one of the patient in our study, with suspicion of mononucleosis 25 days prior to the samples collected for our study, had at that time detectable levels of TBEV RNA. At the time, the patient presented with fever and sore throat. This is in agreement with the notion that TBEV are occurring in the bloodstream in the early phase of infection.

CSF samples were not collected from our study patients. Avoiding an unnecessary invasive procedure was likely to increase the chance that most of the few TBE patients admitted to the hospital consented to participate. Using molecular methods on CSF has been reported to have low diagnostic sensitivity in TBE in other studies [20, 21]. It should, however, be emphasized that molecular detection of viral RNA in CSF may have diagnostic value in certain patient cases, e.g. when patients have not developed neutralising antibodies, are immunosuppressed and/or if the disease is severe and progressive.

Our data suggest that molecular detection of TBEV RNA in blood and body fluids as urine and saliva will mostly not be of any diagnostic value in TBE patients with neurological complications and a moderate course of disease where serum IgM or IgG already is established. Uncertain cases may nevertheless be confirmed by antibody testing, either by observing an increase in antibody titre in paired sera or detection of intrathecal TBEV IgM. Further research is required to clarify the utility of TBEV RNA detection for the diagnostics and monitoring of TBE in particular disease cases, especially for those patients that are negative for IgM and IgG serology with clinical symptoms of viral infections. Viral RNA is useful in surveillance and phylogenetic studies, though not for diagnostic purposes in later stages of disease, at least in immunocompetent patients.
